# Simultaneous Determination of 20 Nitrogen-Containing Heterocyclic Compounds in Soil by Supercritical Fluid Chromatography–Tandem Mass Spectrometry

**DOI:** 10.3390/molecules30061236

**Published:** 2025-03-10

**Authors:** Sergey A. Vakhrameev, Denis V. Ovchinnikov, Nikolay V. Ul’yanovskii, Dmitry S. Kosyakov

**Affiliations:** Laboratory of Environmental Analytical Chemistry, Core Facility Center “Arktika”, Northern (Arctic) Federal University, Arkhangelsk 163002, Russia; v.vahrameev@narfu.ru (S.A.V.); n.ulyanovsky@narfu.ru (N.V.U.); d.kosyakov@narfu.ru (D.S.K.)

**Keywords:** imidazole, pyrazole, triazole, pyridine, supercritical fluid chromatography, tandem mass spectrometry, soil

## Abstract

Nitrogen-containing heterocyclic compounds (NHCs) are common environmental pollutants that need to be monitored due to their high toxicity. Typically, gas or liquid chromatography combined with mass spectrometric detection is used for this task. However, many NHCs are highly polar compounds, which can cause difficulties when using these methods. On the other hand, supercritical fluid chromatography is well-established in the analysis of polar compounds and could provide an alternative to conventional techniques. The presented work proposes an approach to the simultaneous determination of 20 NHCs in soils by supercritical fluid chromatography–tandem mass spectrometry with the limits of quantification in the range 0.08–1.23 mg kg^−1^. The separation is carried out in gradient mode on a cyanopropyl stationary phase in 6 min. The approach was validated and tested on real objects—peat and sandy soils contaminated with rocket fuel transformation products.

## 1. Introduction

Nitrogen-containing heterocyclic compounds (NHCs) are a broad group of compounds that include pyridines, pyrazoles, triazoles, imidazoles, pyrazines and their derivatives. These compounds play an important role in many areas of human life, including agricultural [1,2], pharmaceutical [3,4] or metallurgical [5,6] industries. At the same time, NHCs may be hazardous for living organisms. For example, high concentrations of pyridine in soil caused severe toxicity to earthworms *Eisenia fetida*, inducing oxidative stress and various damaging effects, including lipid damage, DNA injury and histopathological damage [7]. Imidazoles demonstrated a significant toxic effect on aquatic organisms *Daphnia magna*, mainly highlighted by the variation in reproductive parameters [8]. Studies conducted on other aquatic organisms (*Danio rerio*) have shown the occurrence of metabolic disruptions upon triazole exposure [9]. It is worthwhile to note that simple NHCs, like pyridine or pyrazole, are not highly toxic for most organisms; however, each ring substituent can significantly affect their properties, including toxicity [10]. All of the above necessitates monitoring of NHCs in the environment.

The release of NHCs into the environment can occur in various ways, either naturally or due to human impact. On the one hand, NHCs are produced by burning peat and biomass during forest wildfires [11,12], and on the other hand, coal processing, oil production, or waste from the chemical and pharmaceutical industries [13,14]. It is worth mentioning such phenomenon as secondary pollution as a consequence of chemical or biochemical degradation of fungicides, pesticides, insecticides and pharmaceuticals in the environment with the release of NHCs from their structure. For example, propiconazole in soil degrades over time to form 1,2,4-triazole [15].

One of the anthropogenic NHCs sources is space and rocket activity since many countries, including China, Kazakhstan and Russia, use unsymmetrical dimethylhydrazine (UDMH) as rocket fuel [16]. Fuel tanks and engines of the burned-out rocket stages may contain large amounts of residual fuel, which can eventually spill into the environment during the crash landing [17]. UDMH is highly toxic and dangerous itself, and it easily reacts to form a huge number of even more hazardous transformation products, for example, carcinogenic N-nitrosodimethylamine (NDMA). As for heterocyclic compounds, the major UDMH transformation product is 1-methyl-1,2,4-triazole, but several pyrazole and imidazole derivatives were also found [18,19].

Due to the great variety of NHCs, their toxicological properties occurring even at low concentrations, and the complexity of the analyzed matrices, the methods used for monitoring NHCs should have exceptional selectivity and sensitivity. Therefore, chromatography–mass spectrometric methods are universally used to accomplish such challenges.

As many NHCs are volatile, this has prompted the widespread use of gas chromatography (GC). An undoubted advantage of this method is the possibility to use specific sample preparation methods such as headspace solid phase microextraction (HS SPME) or direct thermal desorption (DTD), which, in combination with mass spectrometric detection (GC-MS), allows for quantification limits of NHCs to be reached in soils at the level of fractions of µg kg^−1^ [20,21,22,23,24,25]. Perhaps the only disadvantage of GC-MS is the long analysis time, which can reach several tens of minutes [20,26]. Also, GC is less efficient for the analysis of polar, thermally unstable or non-volatile compounds.

A common alternative to GC is liquid chromatography (LC). Since many NHCs are polar compounds, they are poorly retained on reversed-phase octadecyl sorbents; therefore, mixed-mode [27], zwitterionic [28] or ion-exchange [29,30] stationary phases are used. Some works show the possibility of using porous graphitized carbon as a stationary phase [31]. The mobile phase is usually a mixture of an organic solvent (methanol, acetonitrile) and an aqueous buffer solution; precise pH control of the eluent is strongly required. Among the popular methods of soil sample preparation are acid extraction [29], Soxhlet extraction [31], steam distillation from a strongly alkaline medium [30] and accelerated extraction with a subcritical solvent [28]. LC-MS methods are less sensitive compared to GC-MS and allow the NHCs content in soil to be determined at the level of several µg kg^−1^ [28,29].

We suggest that supercritical fluid chromatography (SFC) may be a promising method for the determination of such compounds. This method is an alternative to LC in the analysis of polar compounds because it allows the use of polar stationary phases, thus realizing the normal-phase separation mode. SFC has been widely used in the analysis of isomers [32,33] and polar compounds [34,35,36]. Nevertheless, we could not find any work devoted to the determination of NHCs in soils by SFC-MS/MS.

Since soil contamination by rocket fuel is an important problem for the countries using ground-based falling regions of burned-out rocket stages (Kazakhstan, Russia) [37], the aim of this work was to develop an approach to the simultaneous determination of nitrogen-containing heterocyclic compounds, which are transformation products of unsymmetrical dimethylhydrazine, by supercritical fluid chromatography–mass spectrometry.

## 2. Results and Discussion

### 2.1. Screening of SFC Stationary Phases and Optimization of Separation Conditions

The basic factor determining the efficiency of chromatographic separation is the stationary phase used, so the first step of the work was column screening. Since the polarity and acid–base properties of the analytes vary over a wide range (Table S1), the selection of the optimal stationary phase was a non-trivial task. The behavior of the analytes on various polar sorbents was investigated, including bare silica gel (BEH), silica gel with cyanopropyl (HSS Cyano), ethylpyridine (BEH 2-EP), pentafluorophenyl (CSH FP) and aminopropyl (NH_2_-RP) groups. In addition, we used an unendcapped octadecyl phase HSS C18 SB, which provides increased retention of polar compounds due to interactions with residual silanol groups.

In preliminary experiments, when pure methanol was used as a co-solvent, excessive interaction with stationary phases was observed for imidazole derivatives, resulting in long retention times and chromatographic peak broadening. A possible explanation could be the protonation of imidazole basic molecules (pKa ≥ 7) in a weakly acidic CO_2_-methanol mixture medium characterized by a pH level of around 5 [38]. Therefore, secondary ionic interactions could occur between the positively charged imidazole molecules and the negatively charged residual silanols. On the other hand, this can be explained by the larger dipole moment of imidazole compared to pyrazole, 1,2,4-triazole and dimethylpyridines [39,40]. Based on the above, methanol with the addition of ammonium formate (AmF), which is often used in the basic compounds analysis to block residual silanol groups on the surface of sorbents [41,42], was used as a co-solvent in further work.

When using an eluent containing 5% methanol (10 mM AmF), the tested stationary phases generally exhibit a close elution order of analytes: methylpyridines < pyrazoles < triazoles < imidazoles (Table 1), indicating similar retention mechanisms.

The retention of methylpyridines on pentafluorophenyl and octadecyl phases is slightly stronger than that of pyrazoles. In the case of the PFP column, this can be explained by a more significant contribution of π–π interactions to the retention. In the second case, increased methylpyridines retention may be due to the nonspecific van der Waals interactions since, based on log *p* values (Table S1), methylpyridines are less polar than pyrazoles. It is also worth noting the decreased elution times of the 1-methyl derivatives (1-methylpyrazole, 1-methyl-1,2,4-triazole and 1-methylimidazole), which indicates a significant contribution of hydrogen bonds to the retention mechanism.

Even in the presence of ammonium formate, the 1H-Imidazole derivatives on all stationary phases eluted much later than the others. This is especially evident for sorbents enriched with silanol groups (BEH and HSS C18 SB), where several imidazoles did not elute in the allotted time of 15 min.

Since the mass spectrometry method is used, the possibility of chromatographic separation of isomers with identical mass becomes critical. In the case of 1H-imidazole and 1,2,4-triazole derivatives, all the selected stationary phases provided efficient separation; however, the separation of pyridine and 1H-pyrazole derivatives proved to be more challenging. Only the pentafluorophenyl and cyanopropyl phases were able to successfully separate isomers of these classes of compounds. The latter was chosen for further experiments as it allows for higher selectivity values to be achieved (Table 2).

As the next step, the influence of the additive type on analyte retention was studied. Apart from ammonium formate, formic acid and water with concentrations in methanol of 0.1% (*v*/*v*) and 5% (*v*/*v*), respectively, were tested as additives. The use of these additives had a significant effect on the retention of dimethyl- and trimethylpyridines, as well as imidazole derivatives (Figure S1). For these compounds, a significant increase in retention times was observed, as well as a deterioration of the chromatographic peak shapes. Since these compounds have a higher pKa values than the rest (Table S1), a possible explanation could be protonation in an acidified mobile phase medium [38]. Therefore, we decided to keep ammonium formate as an additive.

**Figure 1:**
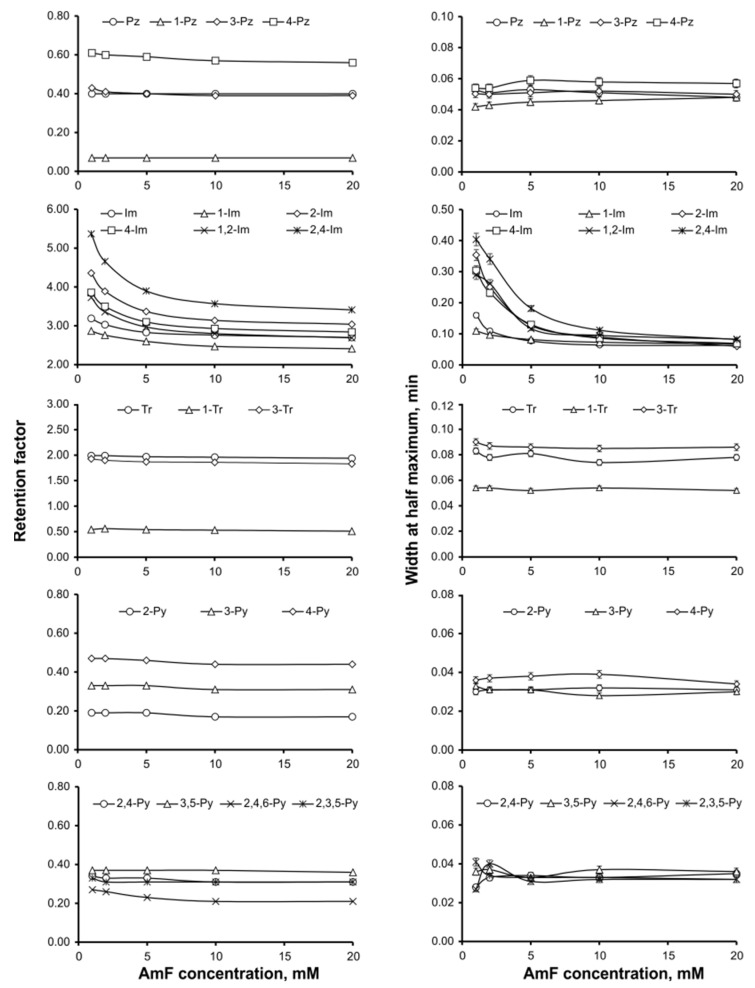
Influence of ammonium formate (AmF) concentration on the retention factor and chromatographic peak width.

The effect of salt additive concentration on analyte retention was studied in the range from 1 to 20 mmol L^−1^ (Figure 1). According to the data obtained, the greatest effect was observed for 1H-imidazole derivatives: with increasing ammonium formate concentration, the retention time and peak width decreased markedly, possibly due to the blocking of residual silanol groups [42,43]. The most intensive changes were observed at up to 10 mmol L^−1^, and further increase of AmF concentration was less effective. Based on the results obtained, the highest studied value, namely 20 mmol L−^1^, was chosen as the final one.

Temperature and back pressure are important factors for SFC, so their influence on analyte retention was studied in the range 10–55 °C and 110–170 bar, respectively. However, in this particular case, varying these parameters did not significantly affect the selectivity and separation efficiency. As a result, intermediate values of 40 °C and 130 bar were chosen as the final.

**Figure 2:**
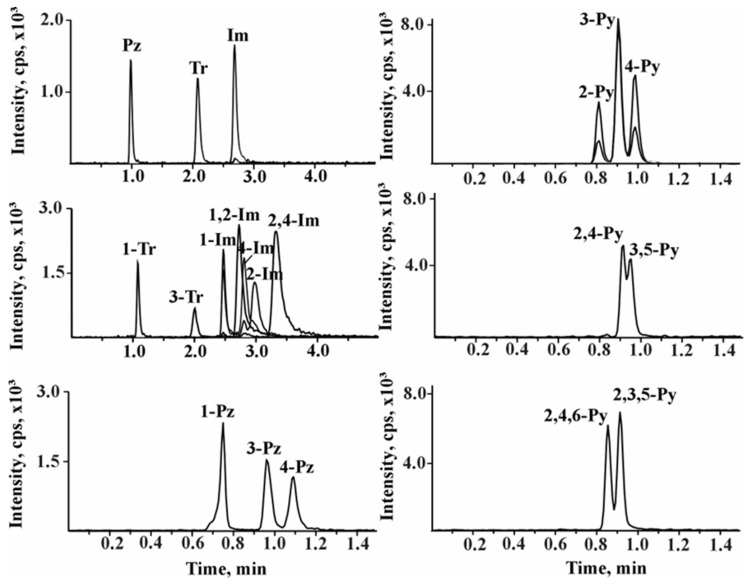
Reconstructed chromatograms of NHCs standard mixture obtained under optimal conditions during the HSS Cyano stationary phase.

Based on all the experiments performed, the following analysis conditions were selected as optimal: HSS Cyano column, flow rate—1.30 mL/min, co-solvent—methanol with 20 mM ammonium formate, column temperature—40 °C, back pressure—130 bar. A gradient elution program was also developed to achieve acceptable separation: 3% MeOH for 0.9 min, increasing to 20% MeOH by 1.5 min and keeping 20% MeOH for 2.5 min. This was followed by equilibration at 3% MeOH for 2 min. Thus, the total analysis time was 6 min. The reconstructed chromatograms of the standard mixture are shown in Figure 2.

### 2.2. Optimization of Mass Spectrometry Detection

**Figure 3:**
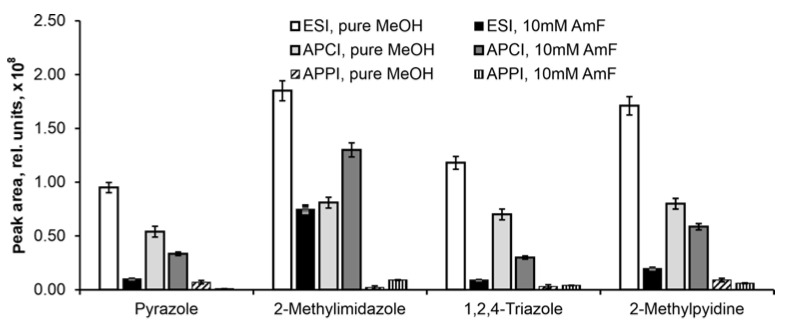
Influence of ion source on ionization efficiency.

The ion source type plays a major role in ion generation efficiency and, therefore, affects the sensitivity of the whole method. During stationary phase screening and mobile phase optimization, we observed a significant suppression of analyte ionization in the presence of ammonium formate during electrospray ionization. Therefore, we conducted an additional experiment to evaluate the effect of ammonium formate on the ionization efficiency of analytes using different types of ionization: electrospray ionization (ESI), atmospheric pressure chemical ionization and photoionization (APCI and APPI, respectively). One representative from each class of compounds was selected, namely pyrazole, 2-methylimidazole, 1,2,4-triazole and 2-methylpyridine (Figure 3).

Of all ion sources, APPI was revealed to have the lowest ion generation ability, even in the presence of dopant (toluene), which may be due to its high flow rate—typical for SFC. The most intense signals were observed under ESI conditions when pure methanol was used, which can be explained by the high proton affinity of analytes. However, when ammonium formate was introduced, the ionization efficiency dropped dramatically. The APCI method demonstrated a slightly lower ionization efficiency compared to ESI when pure methanol was used. The introduction of the additive also led to decreased ionization, but to a lesser extent, whereas 2-methylimidazole, on the contrary, promoted ionization. Since the introduction of ammonium formate is necessary to minimize excessive interactions of nitrogen-containing compounds with polar stationary phases, an APCI source was used in further experiments.

The parameters of the APCI source were optimized to ensure the highest ion generation efficiency: positive mode, corona needle current—4 μA, ion source temperature—300 °C, curtain gas, nebulizer gas and dry gas—20, 50 and 30 psi, respectively. To improve sensitivity and selectivity, the multiple reaction monitoring (MRM) mode was used, and the transition conditions—declustering potential (DP) and collision energy (CE), were optimized for each compound, as presented in Table 3.

### 2.3. Optimization of Extraction Procedure

To extract analytes from the soil, we used a previously developed approach [23] based on the accelerated solvent extraction (ASE) technique. Initially, this approach was developed for acyclic nitrogen-containing compounds (N-nitrosodimethylamine, N,N-dimethylformamide, formaldehyde dimethylhydrazone, etc.), so it was necessary to evaluate its efficiency towards the heterocyclic compounds. For this purpose, a known amount of analytes was added to uncontaminated samples I and III; then, the samples were extracted and analyzed.

The obtained extraction coefficients for peat soil were satisfactory for most of the studied compounds and were in the range 65–115% (Table S2). The exceptions are 1,2,4-triazole and 3-methyl-1,2,4-triazole—with increasing concentration, the extraction efficiency for these compounds decreases to 25 and 38%, respectively. In the case of sandy soil, low extraction efficiencies were also observed for 1,2,4-triazole and 3-methyl-1,2,4-triazole, as well as for imidazole derivatives (Table S3). However, the use of a 9/1 acetonitrile–water mixture instead of pure acetonitrile solved this problem, resulting in extraction efficiencies in the range of 70–115%. Some efficiencies exceeding 100% are observed at low concentrations close to the quantification limit and can be explained by the increased error of the analysis in this concentration range.

### 2.4. Validation of the Developed Method

For all studied compounds, the calibration dependencies were linear within wide concentration ranges (three orders of magnitude) and could be described by the equation of the form “y = a · x” with a correlation coefficient (R^2^) of more than 0.999 (Table 4).

Intra-day and inter-day repeatability were evaluated on standard solutions at three concentration levels. Even at low concentrations, there were no significant deviations (RSD > 15%) (Table S4). The matrix effect was evaluated by a spike recovery test on three concentration levels using soil extracts that did not contain the studied analytes. The obtained spike recoveries (Table S5) were in the range 77–107% for sandy soil and 70–116% for peaty soil. However, a significant ionization suppression of 1,2,4-triazole was observed for the peat soil extract (25–42%).

The approach developed seems to be less sensitive compared to the known GC-MS and HPLC-MS techniques (Table 5) but is superior to them in terms of analysis speed. Another advantage of this approach is the possibility of simultaneous determination of different classes of compounds. Although HPLC methods for the determination of pyridine [44] or imidazole [45] isomers are found in the literature, we were unable to find an approach that allowed the determination of a large number of NHCs of different classes in a single assay. The GC method, which is characterized by higher selectivity, can handle this task [19,26], but such analysis takes much more time.

Thus, this work demonstrates the applicability of the SFC method for the simultaneous determination of NHCs in soils and can be a first step towards further development of this approach.

### 2.5. Analysis of Real Samples

**Figure 4:**
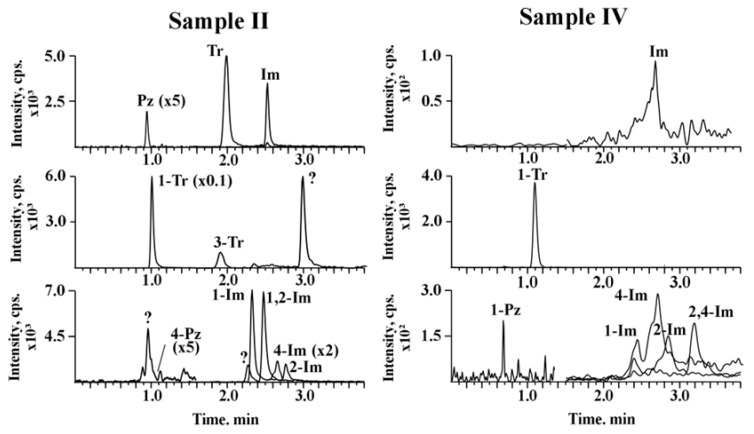
Reconstructed chromatograms of contaminated soil extracts.

The developed approach was tested on the soils contaminated with UDMH, which were collected from the rocket stage fall sites. The obtained chromatograms of soil extracts and concentrations of identified analytes are presented in Figure 4 and Table 6, respectively.

The predominant component in the extracts of both soil types is 1-methyl-1,2,4-triazole, one of the main transformation products of UDMH [22,26], while its content in the sandy soil is an order of magnitude higher. Also, pyrazole and imidazole derivatives were detected in both samples. It is worthwhile to note the presence of foreign peaks (marked with “?”) on the chromatograms of sandy soil extracts. These compounds have ionic transitions corresponding to methylpyrazoles (83.1 → 56.1), methyltriazoles (84.1 → 57.1) and dimethylimidazoles (97.1 → 56.2). However, the peaks of the unidentified compounds are well-separated from analyte peaks and do not disturb the determination.

## 3. Materials and Methods

### 3.1. Analytes, Reagents and Materials

Ultra-high purity carbon dioxide (99.99%, Cryogen, Aramil, Russia) and methanol (HPLC Gradient Grade, Khimmed, Moscow, Russia) were used as mobile phase components. Ammonium formate (10 mol L^−1^ solution in water, Sigma-Aldrich, St. Louis, MO, USA), ammonium hydroxide (28–30% solution, Sigma-Aldrich, St. Louis, MO, USA) and ultrapure Type I Milli-Q water (18.2 MΩ cm) were used as additives. Barium hydroxide octahydrate (pure, Panreac, Barcelona, Spain) and sulfuric acid (extra pure, Komponent-Reactiv, Moscow, Russia) were used in the sample preparation procedure. Toluene (extra pure, Khimmed, Moscow, Russia) was used as a dopant for APPI experiments.

**Figure 5:**
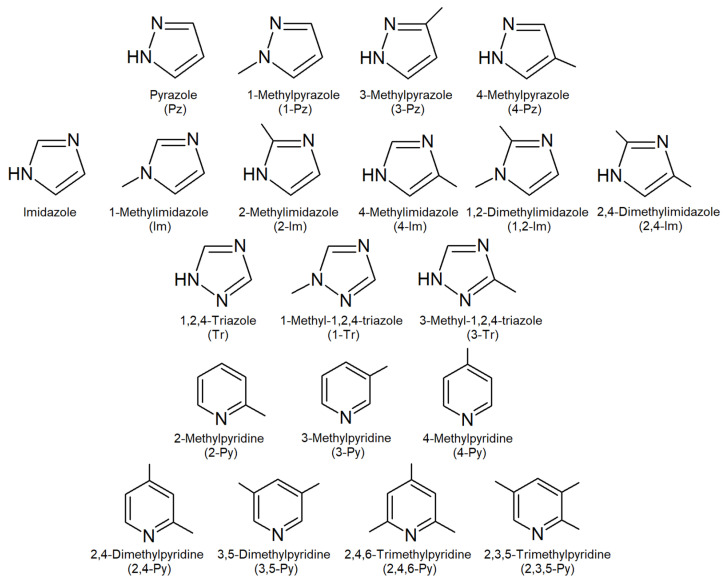
Chemical structures of analytes.

The following nitrogen-containing heterocyclic compounds were chosen as target analytes: 1H-Pyrazole (Pz), 1-Methylpyrazole (1-Pz), 3-Methylpyrazole (3-Pz), 4-Methylpyrazole (4-Pz), 1H-Imidazole (Im), 1-Methylimidazole (1-Im), 2-Methylimidazole (2-Im), 4-Methylimidazole (4-Im), 1,2-Dimethylimidazole (1,2-Im), 2,4-Dimethylimidazole (2,4-Im), 1,2,4-Triazole (Tr), 1-Methyl-1,2,4-triazole (1-Tr), 3-Methyl-1,2,4-triazole (3-Tr), 2-Methylpyridine (2-Py), 3-Methylpyridine (3-Py), 4-Methylpyridine (4-Py), 2,4-Dimethylpyridine (2,4-Py), 3,5-Dimethylpyridine (3,5-Py), 2,4,6-Trimethylpyridine (2,4,6-Py) and 2,3,5-Trimethylpyridine (2,3,5-Py). All compounds with a purity higher than 98% were purchased from Sigma-Aldrich (St. Louis, MO, USA). Their chemical structures and some physico-chemical properties are shown in Figure 5 and Table S1, respectively.

Stock analyte solutions with a concentration of 2 g L^−1^ in methanol were prepared from accurately weighed portions of pure compounds and stored at −20 °C for no more than one week. Working solutions with a concentration of 2 mg L^−1^ for separation optimization and 20 mg L^−1^ for mass spectroscopic detection optimization, as well as calibration solutions, were prepared by serial dilution of the stock solution with acetonitrile/water mixture (9:1 *v*/*v*) and used immediately after preparation.

### 3.2. Real Objects and Sample Preparation

The following samples were used for validation and approbation of the developed approach:

− Sample I—river sand sampled in the bed of the Northern Dvina River (Arkhangelsk region, Russia);
− Sample II—sandy soil collected at the rocket stage fall site (Kazakhstan);
− Sample III—peat soil sampled far from the rocket stage fall (Arkhangelsk region, Russia);
− Sample IV—peat soil collected at the rocket stage fall site (Arkhangelsk region, Russia).

The selected samples were stored in a freezer at −20 °C and defrosted at room temperature before extraction.

Pressurized liquid extraction of soil samples was performed using the ASE-350 accelerated solvent extraction system (Dionex, Sunnyvale, CA, USA) in accordance with the previously developed approach [23].

To extract the target analytes from sandy soil, a sample of 1 g mass (in terms of absolute dry matter) was placed in a 10 mL steel extraction cell. Pure acetonitrile and acetonitrile/water mixture (9:1 *v*/*v*) were used as the extractant. The extraction was carried out at a pressure of 100 atm and a temperature of 100 °C with two extraction cycles—the duration of one cycle was 10 min.

For the extraction of target analytes from peat soil, an average sample of 5 g (0.55 g absolute dry matter) was thoroughly mixed with 1.25 g (2.5-fold excess) of barium hydroxide, and the mixture was introduced into a 10 mL steel extraction cell. An acetonitrile/water mixture (9:1 *v*/*v*) was used as the extractant. The extraction was carried out at a pressure of 100 atm and a temperature of 100 °C in two extraction cycles with a duration of one 10 min cycle. To remove dissolved barium hydroxide from the extract, the obtained extract was neutralized by introducing a 1 mol L^−1^ solution of sulfuric acid dropwise to pH 3–5.

The volume of soil extracts obtained was about 24 mL. The whole extraction time was 30 min. The obtained extracts were centrifuged (14,000 rpm, 10 min), filtered through a nylon membrane with a pore size of 0.22 μm and introduced into the chromatographic system. The extract of sample II (contaminated sand) was diluted 10-fold with a 9:1 *v*/*v* acetonitrile–water mixture before analysis due to its high analyte concentration.

### 3.3. Supercritical Fluid Chromatography–Tandem Mass Spectrometry

The separation was performed using a chromatography–mass spectrometry system consisting of an Acquity UPC^2^ SCF chromatograph (Waters, Milford, OH, USA) including carbon dioxide and co-solvent pumps, autosampler, column thermostat, back pressure controller and tandem mass spectrometer with a 3200 QTrap linear ion trap (AB Sciex, Concord, ON, Canada). The additional make-up co-solvent, which prevented analyte precipitation due to eluent decompression, was introduced using an Ultimate 3000 RS Pump (Thermo Fisher Scientific, Waltham, MA, USA).

The following chromatographic columns were tested during stationary phase screening:

− Acquity UPC^2^ HSS Cyano, 150 × 3.0 mm, 1.8 µm (Waters, USA);
− Acquity UPC^2^ BEH, 150 × 3.0 mm, 1.7 µm (Waters, USA);
− Acquity UPC^2^ BEH 2-EP, 150 × 3.0 mm, 1.7 µm (Waters, USA);
− Acquity UPC^2^ CSH Fluoro-Phenyl, 150 × 3.0 mm, 1.7 µm (Waters, USA)
− Acquity UPC^2^ HSS C18 SB, 150 × 3.0 mm, 1.7 µm (Waters, USA);
− Nucleodur NH_2_-RP, 125 × 2.0 mm, 3.0 µm (Macherey-Nagel, Duren, Germany).

Stationary phase screening experiments were performed under the following conditions: flow rate 1.3 mL/min (except for Nucleodur NH_2_-RP—2.5 mL/min, due to larger particle size), 95% CO_2_: 5% MeOH (10 mmol L^−1^ ammonium formate), temperature 40 °C, back pressure 130 bar, injection volume 2.0 μL, make-up solvent—methanol, 0.10 mL/min.

Mass spectrometric detection was performed in positive mode using electrospray ionization (ESI), atmospheric pressure chemical ionization (APCI), and atmospheric pressure photoionization (APPI).

The efficiency of ion sources was compared at the following parameters: capillary voltage for ESI was 4.5 kV, corona needle current for APCI was 4.0 μA, source voltage for APPI was 750 V; streams of curtain gas (nitrogen), nebulizing and drying gases (air) in the ion source was 20, 20 and 40 psi, respectively. The APPI dopant was toluene, which was fed directly into the source at a rate of 0.1 mL/min. One representative from each class of analyzed compounds was selected for ion source screening: pyrazole, 2-methylimidazole, 1,2,4-triazole and 2-methylpyridine. All experiments were performed without the use of a chromatographic column by introducing 2 μL of test compound solutions into the SFC-MS system.

### 3.4. Method Validation

The limits of determination (LOD) and quantification (LOQ) were calculated using a signal-to-noise ratio (S/N) criteria of 3 and 10, respectively. The matrix effect was estimated by the spike recovery test. A known amount of analytes at three concentration levels was added to the extracts of sandy and peaty soil, initially containing no NHCs (samples I and III). The resulting solutions were prepared as described in Section 3.2 and then analyzed in three replicates.

The intra-day precision (RSD) was estimated at three concentration levels (LOQ, 10 × LOQ, 100 × LOQ) in a series of consecutive chromatographic analyses of the standard solutions (*n* = 10). The inter-day precision was determined in the same manner within 48 h (*n* = 20).

The content of NHCs in real samples and method quantification limits (mg kg^−1^) were calculated using the following formula:

$$X\;=\frac{C\;\cdot \;V}{10\;\cdot \;m\;\cdot \;E\;},$$

where C—NHCs concentration in soil extract (µg L^−1^), V—extract volume (mL), m—sample mass (g) and E—extraction efficiency (%).

## 4. Conclusions

A rapid approach to the simultaneous determination of 20 nitrogen-containing heterocyclic compounds—derivatives of pyrazole, 1,2,4-triazole, imidazole and pyridine—in soil by supercritical fluid chromatography–tandem mass spectrometry was developed. The analytes were separated on cyanopropyl stationary phase in gradient elution mode in 6 min. The limits of quantification are in the range 0.08–0.68 mg kg^−1^ for sandy soil and 0.14–1.23 mg kg^−1^ for peaty soil; the linear concentration range covers three orders of magnitude. The developed approach was validated and tested on real objects—soils contaminated with rocket fuel—unsymmetrical dimethylhydrazine. As far as we know, this is the first time the SFC-MS/MS method has been applied to determine the NHCs in soils. Despite the relatively low sensitivity compared to known GC-MS/MS and LC-MS/MS methods, this work may serve as a basis for further research in this field.

## Figures and Tables

**Table 1:** Retention of analytes on different stationary phases, mobile phase—95% CO_2_: 5% MeOH (10 mM ammonium formate).

Analyte	Retention Coefficient, *k*					
	BEH	HSS C18 SB	CSH FP	HSS Cyano	BEH 2-EP	NH_2_-RP
Pz	0.89	0.74	0.44	0.29	0.41	0.81
1-Pz	0.44	0.36	0.24	0.11	*	*
3-Pz	0.89	0.89	0.47	0.27	0.37	0.75
4-Pz	0.89	0.97	0.51	0.36	0.37	0.75
Im	12.7	10.2	6.30	4.36	3.33	8.94
1-Im	4.84	5.61	3.36	2.04	0.83	1.31
2-Im	**	**	13.3	9.43	5.41	22.8
4-Im	**	17.1	9.24	6.11	3.90	12.8
1,2-Im	19.0	16.4	6.11	3.87	1.34	2.69
2,4-Im	**	**	**	15.5	7.09	37.3
Tr	2.96	2.39	1.39	0.99	1.24	3.31
1-Tr	1.11	1.30	0.64	0.40	0.24	0.38
3-Tr	3.24	2.71	1.63	0.89	1.13	2.38
2-Py	0.53	0.94	0.59	0.19	0.24	0.19
3-Py	0.53	0.94	0.67	0.30	0.24	0.19
4-Py	0.64	1.13	0.86	0.37	0.24	0.19
2,4-Py	0.51	1.04	0.80	0.27	0.24	0.19
3,5-Py	0.61	1.17	0.86	0.33	0.24	0.19
2,4,6-Py	0.59	1.29	0.86	0.23	0.24	0.25
2,3,5-Py	0.70	1.29	0.94	0.27	0.24	0.25

* Eluted at the dead volume. ** Did not elute in the allotted time (15 min).

**Table 2:** Stationary phase selectivity towards critical pairs of isomers, mobile phase—95% CO_2_: 5% MeOH (10 mM ammonium formate).

Isomer Pair	Selectivity, α					
	BEH	HSS C18 SB	CSH FP	HSS Cyano	BEH 2-EP	NH_2_-RP
3-Pz/1-Pz	2.02	2.47	1.96	2.45	12.3	1.75
4-Pz/3-Pz	1.00	1.09	1.09	1.33	1.00	1.00
3-Py/2-Py	1.00	1.00	1.14	1.58	1.00	1.00
4-Py/3-Py	1.21	1.20	1.28	1.23	1.00	1.00
3,5-Py/2,4-Py	1.20	1.13	1.08	1.22	1.00	1.00
2,3,5-Py/2,4,6-Py	1.19	1.00	1.09	1.17	1.00	1.38

**Table 3:** Multiple reaction monitoring (MRM) conditions.

Analyte	Precursor Ion, *m/z*	Product Ion, *m/z*	Declustering Potential, V	Collision Energy, eV
Pz	69	42 (41 *)	30	10
1-Pz	83	56 (42 *)	20	50
3-Pz	83	56 (42 *)	20	50
4-Pz	83	56 (42 *)	30	50
Im	69	42 (41 *)	30	10
1-Im	83	42 (56 *)	30	50
2-Im	83	42 (56 *)	30	50
4-Im	83	56 (42 *)	20	50
1,2-Im	97	56 (42 *)	20	40
2,4-Im	97	56 (42 *)	20	40
Tr	70	43 (42 *)	30	20
1-Tr	84	57 (43 *)	30	30
3-Tr	84	57 (42 *)	30	30
2-Py	94	78 (79 *)	20	40
3-Py	94	78 (79 *)	20	40
4-Py	94	79 (78 *)	40	30
2,4-Py	108	65 (67 *)	30	30
3,5-Py	108	65 (67 *)	30	30
2,4,6-Py	122	79 (106 *)	30	50
2,3,5-Py	122	79 (106 *)	30	50

* Qualifier ion.

**Table 4:** Metrological characteristics of the developed SFC-MS/MS method.

Analyte	*a*	R^2^	Linear Concentration Range, µg L^−1^	LOQ, mg kg^−1^	
				Sandy Soil	Peaty Soil
Pz	0.127	0.9998	40–4000	0.31	0.57
1-Pz	0.491	0.9998	15–1500	0.08	0.14
3-Pz	0.380	0.9999	15–1500	0.10	0.19
4-Pz	0.418	0.9995	10–1000	0.09	0.17
Im	0.189	0.9999	40–4000	0.48	0.88
1-Im	0.280	0.9997	25–2500	0.19	0.34
2-Im	0.503	0.9997	20–2000	0.48	0.88
4-Im	0.685	0.9999	15–1500	0.24	0.44
1,2-Im	0.556	0.9999	20–2000	0.38	0.69
2,4-Im	0.798	0.9998	20–2000	0.35	0.64
Tr	0.134	0.9998	25–2500	0.68	1.23
1-Tr	0.489	0.9998	10–1000	0.25	0.45
3-Tr	0.200	0.9999	10–1000	0.32	0.59
2-Py	0.397	0.9997	20–2000	0.35	0.64
3-Py	0.929	0.9999	10–1000	0.30	0.54
4-Py	1.254	0.9996	10–1000	0.25	0.45
2,4-Py	0.777	0.9999	10–1000	0.12	0.22
3,5-Py	0.930	0.9999	10–1000	0.13	0.24
2,4,6-Py	1.831	0.9999	10–1000	0.12	0.21
2,3,5-Py	2.374	0.9998	10–1000	0.13	0.24

**Table 5:** The comparison of the developed SFC-MS/MS method with known methods.

Analytes	Matrix	Sample Pretreatment	Method	Analysis Time, min	LOQ, µg kg^−1^	Reference
Pz, 1-Pz, 1-Tr (5 UDMH TPs)	sand	vacuum-assisted headspace solid phase microextraction	GC-MS	23	0.56–12	[20]
2-Py, 2,6-Py, 2,4,6-Py, 1-Pz, 1-Im (15 UDMH TPs)	sand	direct thermal desorption	GC-MS/MS	16	1.0–7.7	[21]
2-Py, 2,6-Py, 2,4,6-Py, 1-Pz, 1-Im (15 UDMH TPs)	loamy soil	direct thermal desorption	GC-MS/MS	16	0.8–50	[22]
3-Py, Tr, 1-Tr, Im, 1-Im, 1-Pz, 3-Pz (22 UDMH TPs)	sand, loamy soil	pressurized liquid extraction, solid phase microextraction	GC-MS/MS	57	0.7–1000	[26]
1-Tr (UDMH + 6 TPs)	peaty soil	pressurized liquid extraction	HPLC-MS/MS	9	132	[28]
1-Tr (UDMH + 6 TPs)	peaty soil	acid extraction	HPLC-MS/MS	20	12.6	[29]
20 UDMH TPs	sand, peaty soil	pressurized liquid extraction	SFC-MS/MS	6	80–680, 140–1230	This work

**Table 6:** Measured analyte content in real samples.

Analyte	Content, mg kg^−1^	
	Sample II (Sand)	Sample IV (Peat)
Pz	2.17 ± 0.11	<LOD
1-Pz	<LOD	1.26 ± 0.10
3-Pz	<LOD	<LOD
4-Pz	0.16 ± 0.01	<LOD
Im	10.9 ± 0.7	1.66 ± 0.06
1-Im	24.8 ± 1.5	1.61 ± 0.10
2-Im	3.73 ± 0.25	1.08 ± 0.05
4-Im	0.91 ± 0.05	0.98 ± 0.03
1,2-Im	9.90 ± 0.62	0.93 ± 0.04
2,4-Im	<LOD	<LOD
Tr	39.0 ± 2.4	<LOD
1-Tr	101 ± 8	9.52 ± 0.83
3-Tr	5.56 ± 0.34	<LOD
2-Py	<LOD	<LOD
3-Py	<LOD	<LOD
4-Py	<LOD	<LOD
2,4-Py	<LOD	<LOD
3,5-Py	<LOD	<LOD
2,4,6-Py	<LOD	<LOD
2,3,5-Py	<LOD	<LOD

## Data Availability

All supporting data can be obtained from the corresponding author upon formal request.

## Supplementary Materials

The following supporting information can be downloaded at: https://www.mdpi.com/article/10.3390/molecules30061236/s1, Figure S1: Influence of additive type on the retention of methylpyridines and methylimidazoles; Table S1: Physico-chemical properties of analytes, according to PubChem and ChemSpider databases; Table S2: Extraction efficiency for peat soil; Table S3: Extraction efficiency for sandy soil; Table S4: Intra-day and inter-day repeatability; Table S5: Matrix effect evaluation.
