# Supplementary material for: Simultaneous Determination of 20 Nitrogen-Containing Heterocyclic Compounds in Soil by Supercritical Fluid Chromatography–Tandem Mass Spectrometry
Source: Molecules. 2025 Mar 10;30(6):1236. doi: 10.3390/molecules30061236 (PMC11945050; doi:10.3390/molecules30061236)
Supplement: Supplementary file 1 [file molecules-30-01236-s001.zip › molecules-3488057-supplementary.pdf]

Supplementary Materials

Simultaneous determination of 20 nitrogen-containing heterocyclic compounds in soil by supercritical fluid chromatography – tandem mass spectrometry

Sergey A. Vakhrameev, Denis V. Ovchinnikov*, Nikolay V. Ul'yanovskii and Dmitry S. Kosyakov

Laboratory of Environmental Analytical Chemistry, Core Facility Center "Arktika", Northern (Arctic) Federal University, Arkhangelsk 163002, Russia
**Correspondence: d.ovchinnikov@narfu.ru*

Contents

Figure S1: Influence of additive type on the retention of methylpyridines and methylimidazoles.

Table S1: Physico-chemical properties of analytes, according to PubChem and ChemSpider databases.

Table S2: Extraction efficiency for peat soil.

Table S3: Extraction efficiency for sandy soil.

Table S4: Intra-day and inter-day repeatability.

Table S5: Matrix effect evaluation.

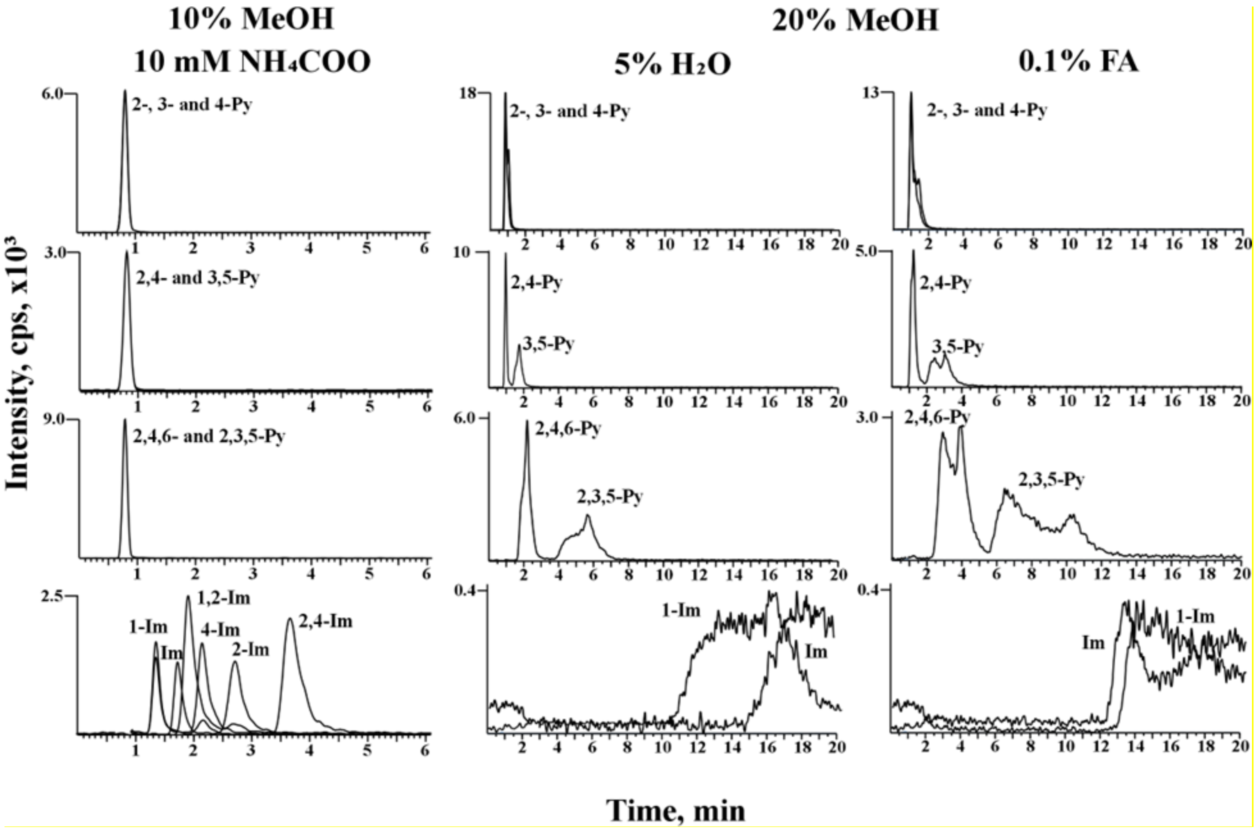

Figure S1. Influence of additive type on the retention of methylpyridines and methylimidazoles.

Table S1. Physico-chemical properties of analytes, according to PubChem and ChemSpider databases.

Analyte	CAS	Formula	Molecular weight	pKa	log P
Pyrazole (Pz)	288-13-1	$C_3H_4N_2$	68.1	2.48	0.26
1-Methylpyrazole (1-Pz)	930-36-9	$C_4H_6N_2$	82.1	2.04	n.a.
3-Methylpyrazole (3-Pz)	1453-58-3	$C_4H_6N_2$	82.1	3.56	n.a.
4-Methylpyrazole (4-Pz)	7554-65-6	$C_4H_6N_2$	82.1	3.09	-0.90
Imidazole (Im)	288-32-4	$C_3H_4N_2$	68.1	6.95	-0.08
1-Methylimidazole (1-Im)	616-47-7	$C_4H_6N_2$	82.1	6.95	-0.21
2-Methylimidazole (2-Im)	693-98-1	$C_4H_6N_2$	82.1	7.86	-0.37
4-Methylimidazole (4-Im)	822-36-6	$C_4H_6N_2$	82.1	7.55	-0.30
1,2-Dimethylimidazole (1,2-Im)	1739-84-0	$C_5H_8N_2$	96.1	n.a.	n.a.
2,4-Dimethylimidazole (2,4-Im)	930-62-1	$C_5H_8N_2$	96.1	8.38	n.a.
1,2,4-Triazole (Tr)	288-88-0	$C_2H_3N_3$	69.1	2.30	-0.58
1-Methyl-1,2,4-triazole (1-Tr)	6086-21-1	$C_3H_5N_3$	83.1	3.20	-0.86
3-Methyl-1,2,4-triazole (3-Tr)	7170-01-6	$C_3H_5N_3$	83.1	3.28	-0.73
2-Methylpyridine (2-Py)	109-06-8	C_6H_7N	93.1	5.96	1.11
3-Methylpyridine (3-Py)	108-99-6	C_6H_7N	93.1	5.63	1.20
4-Methylpyridine (4-Py)	108-89-4	C_6H_7N	93.1	5.98	1.22
2,4-Dimethylpyridine (2,4-Py)	108-47-4	C_7H_9N	107.2	6.63	1.65
3,5-Dimethylpyridine (3,5-Py)	591-22-0	C_7H_9N	107.2	6.15	1.65
2,4,6-Trimethylpyridine (2,4,6-Py)	108-75-8	$C_8H_{11}N$	121.2	7.43	1.88
2,3,5-Trimethylpyridine (2,3,5-Py)	695-98-7	$C_8H_{11}N$	121.2	n.a.	n.a.

n.a. – not available

Table S2. Extraction efficiency for peat soil.

Analyte	Spiked, μg/kg	Found, μg/kg	Efficiency, %
Pz	1.96	1.89 ± 0.10	96 ± 5
	19.6	18.9 ± 1.6	96 ± 8
1-Pz	0.74	0.40 ± 0.03	54 ± 5
	7.40	4.91 ± 0.39	67 ± 5
3-Pz	0.74	0.71 ± 0.08	97 ± 11
	7.40	6.78 ± 0.39	92 ± 5
4-Pz	0.49	0.36 ± 0.04	74 ± 7
	4.90	4.37 ± 0.28	89 ± 6
Im	1.98	1.99 ± 0.24	101 ± 12
	19.7	13.8 ± 0.5	70 ± 3
1-Im	1.23	1.20 ± 0.13	97 ± 1
	12.3	11.5 ± 0.7	94 ± 6
2-Im	1.01	1.09 ± 0.05	108 ± 5
	9.80	8.85 ± 0.37	90 ± 4
4-Im	0.75	0.94 ± 0.07	125 ± 9
	7.40	6.80 ± 0.21	92 ± 3
1,2-Im	1.00	1.10 ± 0.05	110 ± 5
	9.80	9.23 ± 0.37	94 ± 4
2,4-Im	0.98	0.94 ± 0.03	95 ± 3
	9.80	9.22 ± 0.43	94 ± 4
Tr	1.23	0.72 ± 0.08	59 ± 7
	12.3	3.11 ± 0.18	25 ± 2
1-Tr	0.49	0.45 ± 0.04	92 ± 9
	4.90	5.13 ± 0.45	105 ± 9
3-Tr	0.49	0.34 ± 0.02	69 ± 5
	4.90	1.88 ± 0.16	38 ± 3
2-Py	0.49	0.43 ± 0.04	87 ± 9
	4.90	4.74 ± 0.29	97 ± 6
3-Py	0.98	1.06 ± 0.13	108 ± 13
	9.80	9.95 ± 0.81	101 ± 8
4-Py	0.49	0.45 ± 0.02	92 ± 5
	4.90	4.96 ± 0.34	101 ± 7
2,4-Py	0.49	0.39 ± 0.04	79 ± 9
	4.90	4.58 ± 0.34	93 ± 7
3,5-Py	0.49	0.42 ± 0.05	87 ± 11
	4.90	5.00 ± 0.42	102 ± 9
2,3,5-Py	0.49	0.56 ± 0.06	115 ± 12
	4.90	5.18 ± 0.28	106 ± 6
2,4,6-Py	0.49	0.41 ± 0.03	84 ± 5
	4.90	4.78 ± 0.32	97 ± 7

Table S3. Extraction efficiency for sandy soil.

Analyte	Spiked, mg/kg	Extractant – CH_3CN		Extractant – $CH_3CN : H_2O = 9 : 1$	
		Found, mg/kg	Efficiency, %	Found, mg/kg	Efficiency, %
Pz	1.88	1.89 ± 0.10	101 ± 5	1.89 ± 0.01	101 ± 5
	18.8	20.9 ± 1.8	111 ± 10	17.7 ± 1.5	95 ± 8
1-Pz	0.70	0.60 ± 0.05	86 ± 7	0.49 ± 0.04	70 ± 6
	7.00	7.31 ± 0.59	104 ± 8	7.21 ± 0.58	103 ± 8
3-Pz	0.70	0.74 ± 0.08	106 ± 12	0.76 ± 0.09	108 ± 12
	7.00	8.38 ± 0.48	119 ± 7	6.32 ± 0.36	90 ± 5
4-Pz	0.47	0.46 ± 0.05	98 ± 10	0.45 ± 0.04	96 ± 10
	4.70	4.84 ± 0.31	103 ± 7	4.42 ± 0.28	94 ± 6
Im	1.88	1.21 ± 0.15	65 ± 8	2.08 ± 0.25	111 ± 14
	18.8	12.0 ± 0.4	64 ± 2	16.4 ± 0.6	88 ± 3
1-Im	1.17	0.81 ± 0.09	69 ± 8	1.14 ± 0.12	97 ± 11
	11.7	9.40 ± 0.59	80 ± 5	9.69 ± 0.60	83 ± 5
2-Im	0.94	0.47 ± 0.02	50 ± 2	0.96 ± 0.05	102 ± 5
	9.40	3.95 ± 0.17	42 ± 2	8.87 ± 0.37	95 ± 4
4-Im	0.70	0.69 ± 0.05	99 ± 7	0.81 ± 0.06	115 ± 8
	7.0	3.82 ± 0.12	54 ± 2	6.17 ± 0.19	88 ± 3
1,2-Im	0.94	0.37 ± 0.02	40 ± 2	0.99 ± 0.05	106 ± 5
	9.40	2.32 ± 0.09	25 ± 1	9.26 ± 0.37	99 ± 4
2,4-Im	0.94	0.39 ± 0.01	41 ± 2	0.92 ± 0.03	98 ± 4
	9.40	4.61 ± 0.21	49 ± 2	8.37 ± 0.39	89 ± 4
Tr	1.17	0.52 ± 0.06	45 ± 5	1.33 ± 0.15	113 ± 13
	11.7	9.18 ± 0.53	78 ± 5	13.2 ± 0.9	113 ± 8
1-Tr	0.47	0.47 ± 0.05	101 ± 10	0.46 ± 0.04	98 ± 9
	4.70	4.98 ± 0.44	106 ± 9	4.57 ± 0.40	98 ± 9
3-Tr	0.47	0.30 ± 0.02	65 ± 5	0.46 ± 0.03	99 ± 7
	4.70	4.48 ± 0.38	96 ± 8	4.60 ± 0.39	98 ± 8
2-Py	0.47	0.48 ± 0.05	102 ± 11	0.39 ± 0.02	83 ± 10
	4.70	5.28 ± 0.32	113 ± 7	7.81 ± 0.50	87 ± 5
3-Py	0.94	0.91 ± 0.11	97 ± 12	0.52 ± 0.05	110 ± 11
	9.40	10.5 ± 0.9	112 ± 9	4.10 ± 0.46	83 ± 7
4-Py	0.47	0.46 ± 0.03	98 ± 5	0.45 ± 0.02	95 ± 5
	4.70	4.81 ± 0.33	103 ± 7	3.79 ± 0.26	81 ± 6
2,4-Py	0.47	0.44 ± 0.05	94 ± 11	0.44 ± 0.05	93 ± 11
	4.7	4.74 ± 0.35	101 ± 8	3.88 ± 0.29	83 ± 6
3,5-Py	0.47	0.50 ± 0.06	107 ± 13	0.47 ± 0.06	101 ± 12
	4.70	5.13 ± 0.43	109 ± 9	4.82 ± 0.40	103 ± 9
2,3,5-Py	0.47	0.38 ± 0.04	81 ± 9	0.46 ± 0.05	99 ± 11
	4.70	4.34 ± 0.23	93 ± 5	4.83 ± 0.26	103 ± 6
2,4,6-Py	0.47	0.45 ± 0.03	97 ± 6	0.48 ± 0.03	103 ± 7
	4.70	4.69 ± 0.31	100 ± 7	5.03 ± 0.33	107 ± 7

Table S4. Intra-day and inter-day repeatability.

Analyte	Concentration, μg/L	Intra-day		Inter-day	
		RSD, %	Accuracy, %	RSD, %	Accuracy, %
Pz	40	4.3	100	5.2	102
	400	7.6	106	8.5	103
	4000	3.8	99.5	6.2	99.3
1-Pz	15	8.4	96.3	9.0	101
	150	4.0	103	8.0	98.7
	1500	6.2	99.2	6.8	101
3-Pz	15	11	107	14	107
	150	5.1	98.6	5.7	98.0
	1500	3.7	96.3	7.2	97.1
4-Pz	10	8.8	106	10.0	102
	100	5.7	96.6	6.4	96.8
	1000	5.9	97.6	7.3	99.5
Im	40	9.8	106	12	105
	400	3.5	104	4.5	103
	4000	4.3	97.3	6.2	99.0
1-Im	25	8.0	99.8	11	104
	250	6.0	98.3	6.5	96.8
	2500	4.1	95.9	5.9	96.6
2-Im	20	4.8	102	6.2	101
	200	4.0	96.9	4.5	95.9
	2000	4.1	96.3	6.6	96.4
4-Im	15	5.8	115	7.2	111
	150	1.8	102	3.1	101
	1500	3.8	95.4	5.9	95.4
1,2-Im	20,0	4.1	100	4.8	103
	200	3.8	97.5	4.0	96.7
	2000	3.0	97.0	5.1	97.9
2,4-Im	20	3.1	99.8	3.6	99.7
	200	4.6	97.1	5.3	96.0
	2000	3.4	96.5	6.3	98.1
Tr	25	8.9	101	11	102
	250	5.8	97.2	6.4	95.9
	2500	3.4	98.4	6.2	98.2
1-Tr	10	9.7	98.8	11	97.5
	100	2.3	100	8.8	94.7
	1000	3.4	98.1	7.8	96.1
3-Tr	10	7.2	101	9.1	102
	100	8.1	96.0	8.6	95.1
	1000	4.1	95.5	6.2	96.2
2-Py	40	9.7	106	10	109
	400	6.1	99.2	7.5	99.6
	4000	5.3	92.5	8.3	97.2
3- Py	10	12	104	13	104
	100	5.3	101	8.1	96.7
	1000	5.7	96.0	6.2	96.2
4- Py	10	5.5	97.7	6.3	100
	100	5.1	97.4	6.9	94.2
	1000	4.8	93.1	7.1	96.1
2,4-Lu	10	8.8	97.9	12	103
	100	4.2	99.4	7.5	95.5
	1000	2.7	96.9	9.4	96.8
3,5- Py	10	9.2	106	12	105
	100	8.4	104	11	102
	1000	4.1	104	7.4	102
2,4,6-Py	10	11	102	13	101
	100	4.5	99.1	5.4	96.8
	1000	3.2	98.2	7.0	97.5
2,3,5-Py	10	5.7	95.7	6.4	95.5
	100	4.0	96.3	6.7	92.2
	1000	4.1	101	6.9	98.5

Table S5. Matrix effect evaluation.

Analyte	Spiked, μg L^{-1}	Peaty soil		Sandy soil	
		Found μg L^{-1}	Recovery, %	Found μg L^{-1}	Recovery, %
Pz	50	46.5 ± 2.0	93.0 ± 4.0	48.6 ± 2.1	97.1 ± 4.2
	500	459 ± 19	91.7 ± 3.8	483 ± 20	96.5 ± 4.0
	5000	3953 ± 99	79.1 ± 2.0	3999 ± 100	80.0 ± 2.0
1-Pz	20	18.0 ± 3.0	90.0 ± 15.0	17.8 ± 2.9	88.8 ± 14.7
	200	176 ± 5	88.1 ± 2.6	180 ± 5	90.0 ± 2.5
	2000	1682 ± 39	84.1 ± 1.3	1871 ± 44	93.6 ± 2.2
3-Pz	20	20.0 ± 3.1	102 ± 15	15.7 ± 2.3	78.3 ± 11.7
	200	182 ± 5	91.1 ± 2.6	168 ± 5	83.8 ± 2.4
	2000	1774 ± 25	88.7 ± 1.2	1816 ± 26	90.8 ± 1.3
4-Pz	20	18.5 ± 1.4	92.3 ± 6.8	17.5 ± 1.3	87.5 ± 6.5
	200	180 ± 5	89.9 ± 2.4	171 ± 5	85.5 ± 2.3
	2000	1639 ± 31	82.0 ± 1.6	1831 ± 35	91.5 ± 1.7
Im	40	46.4 ± 4.5	116 ± 11	42.6 ± 4.0	107 ± 10
	400	421 ± 32	105 ± 8	410 ± 31	103 ± 8
	4000	3866 ± 54	96.7 ± 1.4	4147 ± 58	104 ± 2
1-Im	25	25.5 ± 6.0	102 ± 24	24.1 ± 5.6	96.0 ± 23
	250	225 ± 15	90.2 ± 6.0	224 ± 15	89.8 ± 6.1
	2500	2102 ± 30	84.1 ± 1.2	2540 ± 36	102 ± 1
2-Im	15	14.8 ± 3.2	98.5 ± 15.0	14.8 ± 3.2	98.7 ± 2.1
	150	146 ± 13	97.5 ± 3.1	134 ± 12	89.6 ± 8.0
	1500	1510 ± 21	101 ± 2	1574 ± 22	105 ± 2
4-Im	25	27.4 ± 3.8	110 ± 15	26.2 ± 3.6	104 ± 14
	250	270 ± 8	108 ± 3	234 ± 7	93.4 ± 2.7
	2500	2264 ± 40	90.5 ± 1.6	2540 ± 45	102 ± 2
1,2-Im	30	30.0 ± 3.7	99.5 ± 12.4	28.1 ± 3.5	93.7 ± 11.7
	300	308 ± 10	103 ± 3	271 ± 9	90.4 ± 2.8
	3000	2894 ± 51	96.5 ± 1.7	2894 ± 52	96.5 ± 1.7
2,4-Im	40	38.9 ± 10.5	97.2 ± 26.3	39.8 ± 10.7	99.4 ± 27
	400	423 ± 12	106 ± 3	366 ± 11	91.48 ± 2.7
	4000	4092 ± 58	102 ± 2	4145 ± 59	104 ± 2
Tr	40	10.0 ± 2.2	25.1 ± 5.4	37.8 ± 4.4	77.0 ± 20.2
	400	148 ± 15	36.9 ± 3.8	421 ± 44	105 ± 11
	4000	1681 ± 30	42.0 ± 0.8	3522 ± 62	88.1 ± 1.5
1-Tr	30	29.9 ± 4.2	100 ± 14	30.8 ± 4.4	103 ± 18
	300	272 ± 20	90.7 ± 6.7	278 ± 20	92.8 ± 4.1
	3000	2534 ± 46	84.5 ± 1.5	2813 ± 51	93.8 ± 1.3
3-Tr	25	19.5 ± 3.7	78.0 ± 15.0	24.5 ± 4.5	97.9 ± 11.1
	250	202 ± 8	80.6 ± 3.1	265 ± 10	106 ± 3
	2500	2038 ± 25	81.5 ± 1.0	2677 ± 33	107 ± 6
2-Py	10	10.2 ± 1.1	102 ± 11	10.2 ± 1.6	102 ± 16
	100	110 ± 4	110 ± 4	91.4 ± 3.4	91.4 ± 4.8
	1000	814 ± 49	81.4 ± 4.9	940 ± 57	94.0 ± 1.9
3- Py	10	9.1 ± 1.5	90.9 ± 15	9.9 ± 1.6	99.0 ± 11.0
	100	74.8 ± 3.8	74.8 ± 3.9	92.8 ± 4.8	92.8 ± 3.0
	1000	711 ± 14	71.1 ± 1.4	970 ± 19	97.0 ± 3.6
4- Py	10	9.6 ± 1.0	96.3 ± 10.0	10.7 ± 1.1	107 ± 11
	100	90.5 ± 3.0	90.4 ± 3.0	91.2 ± 3.0	91.2 ± 3
	1000	862 ± 34	86.2 ± 3.4	929 ± 36	93.0 ± 3.6
2,4-Lu	10	9.2 ± 1.8	92.7 ± 18	10.5 ± 2.0	104 ± 20
	100	95.0 ± 5.3	95.0 ± 5.4	92.00 ± 5.2	92.00 ± 5.2
	1000	863 ± 35	86.3 ± 3.5	930 ± 38	93.0 ± 3.8
3,5- Py	10	10.2 ± 1.7	102 ± 17	9.7 ± 1.6	97.1 ± 16.1
	100	88.6 ± 5.4	88.6 ± 5.4	93.4 ± 5.7	93.4 ± 5.7
	1000	886 ± 21	88.6 ± 2.1	893 ± 21	89.3 ± 3.7
2,4,6-Py	10	10.3 ± 1.1	104 ± 11	9.3 ± 1.0	92.9 ± 9.8
	100	87.6 ± 4.0	87.6 ± 4.0	84.8 ± 3.8	84.8± 3.8
	1000	845 ± 12	84.5 ± 1.2	882 ± 12	88.2 ± 1.2
2,3,5-Py	10	10.9 ± 1.0	109 ± 10	11.0 ± 1.0	110 ± 10
	100	92.2 ± 2.8	92.2 ± 2.8	89.0 ± 2.7	89.0 ± 2.7
	1000	860 ± 41	86.0 ± 4.1	926 ± 4	92.6 ± 4.4
